# Tinnitus with a normal audiogram: Relation to noise exposure but no evidence for cochlear synaptopathy

**DOI:** 10.1016/j.heares.2016.12.002

**Published:** 2017-02

**Authors:** Hannah Guest, Kevin J. Munro, Garreth Prendergast, Simon Howe, Christopher J. Plack

**Affiliations:** a Manchester Centre for Audiology and Deafness, University of Manchester, Manchester Academic Health Science Centre, UK; b Audiology Department, James Cook University Hospital, South Tees Hospitals NHS Foundation Trust, Middlesbrough, UK; c Central Manchester University Hospitals NHS Foundation Trust, Manchester, UK; d Department of Psychology, Lancaster University, Lancaster, UK

**Keywords:** Tinnitus, Cochlear synaptopathy, Hidden hearing loss, Auditory brainstem response, Envelope following response, Noise-induced hearing loss, ABR, auditory brainstem response, AN, auditory nerve, EFR, envelope following response, SR, spontaneous rate, TNA, tinnitus with a normal audiogram

## Abstract

In rodents, exposure to high-level noise can destroy synapses between inner hair cells and auditory nerve fibers, without causing hair cell loss or permanent threshold elevation. Such “cochlear synaptopathy” is associated with amplitude reductions in wave I of the auditory brainstem response (ABR) at moderate-to-high sound levels. Similar ABR results have been reported in humans with tinnitus and normal audiometric thresholds, leading to the suggestion that tinnitus in these cases might be a consequence of synaptopathy. However, the ABR is an indirect measure of synaptopathy and it is unclear whether the results in humans reflect the same mechanisms demonstrated in rodents. Measures of noise exposure were not obtained in the human studies, and high frequency audiometric loss may have impacted ABR amplitudes. To clarify the role of cochlear synaptopathy in tinnitus with a normal audiogram, we recorded ABRs, envelope following responses (EFRs), and noise exposure histories in young adults with tinnitus and matched controls. Tinnitus was associated with significantly greater lifetime noise exposure, despite close matching for age, sex, and audiometric thresholds up to 14 kHz. However, tinnitus was not associated with reduced ABR wave I amplitude, nor with significant effects on EFR measures of synaptopathy. These electrophysiological measures were also uncorrelated with lifetime noise exposure, providing no evidence of noise-induced synaptopathy in this cohort, despite a wide range of exposures. In young adults with normal audiograms, tinnitus may be related not to cochlear synaptopathy but to other effects of noise exposure.

## Highlights

• Tinnitus participants matched with controls for age, sex, & audiogram up to 14 kHz.
• Tinnitus participants more noise exposed, despite close audiometric matching.
• No ABR or EFR evidence for cochlear synaptopathy in tinnitus participants.
• No association between ABR or EFR measures and lifetime noise exposure.

## 1 Introduction

Subjective tinnitus – the perception of sound without an acoustic source – is most often associated with hearing loss (Nicolas-Puel et al., 2002, Sanchez et al., 2005). It is widely agreed that these phenomena are related, with hearing loss usually regarded as a trigger for neuroplastic changes in the central auditory system, giving rise to the tinnitus percept. While these central changes differ in the various prevailing neural models of tinnitus, they are generally thought to be provoked by loss of input from the auditory nerve (AN) to central auditory structures (Henry et al., 2014, Schaette, 2014).

Seemingly at odds with this widespread account of tinnitus generation, approximately 8% of tinnitus patients have pure tone audiometric thresholds within the normal range (Barnea et al., 1990, Sanchez et al., 2005). The prevalence of tinnitus with a normal audiogram (TNA) might be taken to indicate that cochlear damage is not a routine requirement of tinnitus generation. However, recent findings in a variety of rodent models have suggested otherwise, by demonstrating that substantial damage to the auditory periphery can occur without affecting cochlear thresholds. Seminal research in mice by Kujawa and Liberman (2009) revealed that carefully titrated noise exposure can lead to immediate and extensive loss of synapses between cochlear inner hair cells and AN fibers, yet leave inner and outer hair cells macroscopically intact. Termed “cochlear synaptopathy”, this primary deafferentation has also been observed in noise-exposed guinea pigs (Lin et al., 2011) and in aging mice without significant noise exposure (Sergeyenko et al., 2013). Crucially, the pathology does not compromise sensitivity to low-level sounds, seemingly due to preferential loss of AN fibers with low spontaneous firing rates (SRs) and high thresholds (Furman et al., 2013). Consistent with low-SR fiber loss, abnormal auditory processing is evident at higher sound levels. Synaptopathic ears exhibit permanent reductions in the amplitude of wave I of the auditory brainstem response (ABR) to tone bursts with moderate-to-high sound levels (Kujawa and Liberman, 2009).

Similar electrophysiological evidence of deafferentation has been reported in humans with TNA. Schaette and McAlpine (2011) recorded ABRs to clicks with high sound levels and demonstrated reductions in wave I amplitude in TNA subjects relative to audiogram-matched controls. The results were interpreted as evidence of deafferentation consistent with cochlear synaptopathy: a “hidden hearing loss” which might resolve the enigma of TNA. The absence of any tinnitus-related reduction in ABR wave V was tentatively attributed to increased central gain in the auditory brainstem, suggested as a mechanism of tinnitus generation. Gu et al. (2012) reported similar findings in subjects with near-normal hearing.

However, the latter study demonstrated significant wave I amplitude reductions only for the highest stimulus level used, 120 dB peSPL, and not for lower levels more comparable with those of Schaette and McAlpine (≤ 100 dB peSPL). Missing ABR data at this high stimulus level led to reduced participant groups with unmatched audiograms at high frequencies (tinnitus had systematically poorer mean thresholds above 8 kHz). This disparity may have accounted for the group difference in ABR amplitude, since wave I is dominated by the responses of high frequency AN fibers (Don and Eggermont, 1978). Schaette and McAlpine's tinnitus and control groups also differed in high frequency sensitivity. Mean 12 kHz threshold was elevated by 3.5 dB in the tinnitus group, and thresholds at even higher frequencies were not reported. Additionally, a recent study by Gilles et al. (2016) found no wave I amplitude reduction in young people with tinnitus, though statistical power was compromised by high measurement variability. Given the growing interest in cochlear synaptopathy in humans, the evidence for its role in tinnitus could benefit from careful confirmation.

Investigation of the condition in living humans is necessarily indirect and requires a sensitive, non-invasive measure. The transient-evoked ABR may offer limited sensitivity to synaptopathy in humans, despite clear correlations with the pathology in rodent models. ABR amplitudes are highly variable, influenced by factors such as head size, cochlear dispersion, and skull thickness (Michalewski et al., 1980, Trune et al., 1988, Don et al., 1994), which might obscure the effects of synaptopathy. Differential ABR measures may minimize the influence of these non-synaptopathic factors (Plack et al., 2016), but recent evidence suggests a more fundamental shortcoming of the ABR. Recordings in gerbils and guinea pigs after ototoxic exposure indicate that AN fibers with the lowest SRs do not contribute to the compound action potential, equivalent to ABR wave I (Bourien et al., 2014). The low-SR fibers affected in animal models of synaptopathy exhibit a somewhat wider range of firing rates than those described by Bourien and colleagues (Furman et al., 2013). Nevertheless, the former exhibit relatively weak onset responses (Taberner and Liberman, 2005), limiting their contribution to the ABR (Shaheen et al., 2015).

In contrast, low-SR fibers surpass high-SR fibers in their synchronization to amplitude-modulated stimuli (Joris et al., 2004). Hence they make robust contributions to the subcortical envelope following response (EFR): a sustained response representing neural synchrony to the envelope of an amplitude-modulated stimulus. Relatively high modulation frequencies are necessary to elicit the subcortical EFR. At lower frequencies, below 80 Hz, responses are dominated by cortical generators (Kuwada et al., 2002). Using EFR stimuli optimized to enhance the contribution from the AN, Shaheen et al. (2015) demonstrated that EFR amplitude afforded greater sensitivity to noise-induced cochlear synaptopathy in mice than ABR amplitude. An additional strategy to enhance the sensitivity of the EFR was devised by Bharadwaj et al. (2015), who reasoned that stimuli with high sound levels and shallow modulations should be weakly encoded in synaptopathic ears, due to saturation of high-SR fibers and consequent reliance on low-SR units. To reduce variability from non-synaptopathic sources that might affect raw EFR amplitude, the researchers computed the slope of the function relating EFR amplitude to stimulus modulation depth. This measure was shown to correlate with behavioral measures of temporal coding and auditory selective attention in audiometrically normal humans, with synaptopathy proposed as a potential underlying cause. Hence carefully designed EFR measures may be of value in the identification of cochlear synaptopathy in humans.

Finally, previous studies associating TNA with evidence of cochlear synaptopathy have not obtained measures of lifetime noise exposure. Indeed, to the authors’ knowledge, no previous study has reported that TNA is associated with elevated noise exposure compared to audiogram-matched controls. It is therefore unclear whether the reported electrophysiological effects in TNA are caused by the same mechanisms demonstrated in rodent models of noise-induced synaptopathy.

The fourfold aims of the present study were: (a) To determine whether TNA is associated with greater lifetime noise exposure; (b) To provide a further test of the hypothesis that TNA is associated with ABR effects consistent with cochlear synaptopathy, controlling for high frequency sensitivity; (c) To determine whether TNA is associated with temporal coding deficits consistent with synaptopathy; (d) To examine the relations between electrophysiological measures of synaptopathy and lifetime noise exposure.

## 2 Material and methods

### 2.1 Participants

Control participants were recruited from the University of Manchester staff and student populations (via poster and on-line advertising) and from the general Manchester population (via on-line advertising). Tinnitus participants were recruited from the same sources, with the addition of patients identified by local audiology services. All participants were required to exhibit bilaterally normal pure tone audiometric thresholds (≤ 20 dB HL at 0.25–8 kHz) and middle ear function (compliance 0.3–1.6 ml; middle ear pressure −50 to +50 daPa). All were without history of head trauma, middle ear surgery, neurological disorder, and ototoxic exposure.

Tinnitus participants (n = 20, female = 10) were aged 25.7 ± 1.3 years (mean ± standard error of the mean). All reported prolonged spontaneous tinnitus that was stable (> 4 months) and non-pulsatile. Tinnitus characteristics are summarized in Table 1. The mean Tinnitus Functional Index (TFI) score was 33 (± 7), which corresponds to “moderate” problems with tinnitus on average (Henry et al., 2016).

**Fig. 1:**
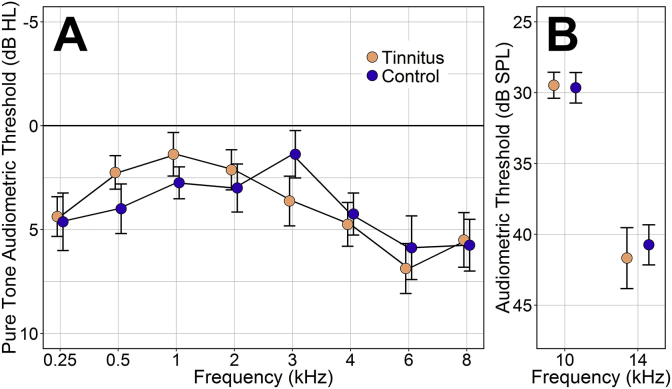
Audiometric thresholds for tinnitus and control groups, presented as group mean ± standard error of the mean. A: Pure tone audiometric thresholds. Groups means differ by < 2.25 dB at all frequencies. B: High frequency thresholds for 1/3-octave narrowband noise using a three-interval, three-alternative, forced-choice paradigm and a two-down, one-up rule. Group means differ by < 1 dB.

Control participants (n = 20, female = 10, mean age = 25.5 ± 1.3 years) were individually matched with tinnitus participants on the basis of age (to within 18 months) and sex. Mean audiometric thresholds were matched between groups to within 2.3 dB at all test frequencies from 0.25 to 14 kHz, after averaging the left and right ear thresholds. At the extended high frequencies (10 and 14 kHz), the group means differed by < 1 dB (Fig. 1).

Sample size was selected to provide 80% power (α = 0.05, one-tailed) to detect the ABR effect size demonstrated by Schaette and McAlpine (2011) for a 100 dB peSPL stimulus. It should be noted that the previous study recruited only female participants, whereas the present study recruited a mixed sex sample, potentially inflating ABR amplitude variability. However, variability from other sources was expected to be reduced (e.g. by use of active electrodes) and this expectation was fulfilled (see 3.2 and 4.2 for post-hoc power analysis).

### 2.2 Noise exposure history

#### 2.2.1 General procedure

Each participant provided a detailed history of lifetime noise exposure via structured interview, based on the procedure described by Lutman et al. (2008). For all exposures estimated to exceed 80 dBA (see 2.2.3), data were gathered on estimated sound level, total duration of exposure, and use of personal hearing protection. The participant provided information first on occupational noise exposure, followed by social noise exposure. The duration of the structured interview ranged from 5 to 45 min. Example noise exposure data for a single participant are given in Table 3 of Supplementary Material.

#### 2.2.2 Determination of activities incurring noise exposure

The participant was asked to recall activities that routinely involved exposure to sound levels ≥ 80 dBA (see 2.2.2). A list of the most common social activities involving noise was provided (given in Lutman et al., 2008). Each activity identified by the participant was marked as an entry in their noise record, and associated information sought on duration and sound level. An activity was treated as a single entry only if it entailed approximately consistent sound levels throughout all exposures. If the sound level varied, then the exposures were broken down into two or more activities (e.g. “loud bars” and “quieter bars” or “metal gigs” and “rock gigs”).

#### 2.2.3 Estimation of sound level

For free-field exposures, sound levels were estimated based on vocal effort required to hold a conversation at a distance of 1.2 m. Reported vocal effort was converted to dBA level using a speech communication table (Lutman et al., 2008; see Table 2 of Supplementary Material). For example, if the participant recalled that it was necessary merely to “raise one's voice” to hold a conversation (rather than “talk very loudly” or “shout”), an estimated level of 87 dBA was selected. Information was also provided on use of personal hearing protection: type, attenuation (if known), and proportion of time worn during each activity. When attenuation was unknown, it was estimated from type of protector (see Lutman et al., 2008).

For exposures incurred through use of personal music players, the participant reported the typical setting of the volume control on their device, expressed as a percentage of the maximum setting. This value was converted to a free-field equivalent output level, based on the output levels measured by Portnuff et al. (2011) across a variety of devices coupled to stock earphones (see Table 2 of Supplementary Material).

#### 2.2.4 Estimation of exposure duration

For a given activity, the participant identified a time period (usually a number of years) during which they had engaged in the activity with approximately uniform regularity. The participant then estimated the number of hours per day, days per week, and weeks per year of exposure during that period, allowing calculation of total hours of exposure. Often, the participant would report having engaged in an activity more frequently during one period than another. Hours of exposure would be calculated for each period separately, then summed. Additionally, where hearing protection had been worn only part of the time, it was necessary to calculate the protected and unprotected exposure durations.

#### 2.2.5 Calculation of units of noise exposure

For each activity in the noise record, duration, level, and protector attenuation were combined to generate units of noise exposure based on the equal energy principle:

        U = 10^(L−A−90)/10^ × T / 2080

where:    U = units of noise exposure
       L = level (dBA)
       A = attenuation of ear protection (dBA)
       T = total exposure time (hours)

The units from all exposures, regardless of whether they occurred in social or occupational settings, were summed to yield the total units of lifetime noise exposure. The resulting measure is linearly related to the total energy of exposure above 80 dBA.

### 2.3 Behavioral testing

Participants were seated in a double-walled sound-attenuating booth, providing responses using a button (pure tone audiometry) or mouse and computer monitor (high-frequency audiometry). Air conduction pure tone audiometric thresholds were obtained in accordance with British Society of Audiology recommended procedures (British Society of Audiology, 2011) at 0.25, 0.5, 1, 2, 3, 4, 6, and 8 kHz, using a GSI Arrow audiometer, TDH-39 supra-aural headphones, and MX-41 ear cushions. High-frequency thresholds were obtained using a three-interval, three-alternative, forced-choice paradigm, with stimuli delivered through Sennheiser HDA 200 circum-aural headphones driven by an E-MU 0202 external audio interface. In order to minimize the influence of threshold microstructure and ear canal resonance, stimuli were 1/3-octave bands of noise centered at 10 and 14 kHz. Steady-state duration was 180 ms, with the addition of 10 ms raised-cosine onset and offset ramps. Stimulus level was varied adaptively using a two-down, one-up rule. Threshold was attained using three initial turnpoints (6 dB step size) and eight subsequent turnpoints (2 dB step size). The stimulus level at the final eight turnpoints was averaged to obtain threshold. Thresholds were obtained for each ear separately and then averaged across ears. Prior to testing, each participant performed a practice run containing at least three turnpoints.

### 2.4 Auditory evoked potentials

#### 2.4.1 General procedure

Participants reclined comfortably with eyes closed in a double-walled sound-attenuating booth. Auditory stimuli were delivered through EARtone 3A insert earphones with mu-metal and aluminum shielding, driven by an Avid FastTrack C400 external audio interface (48 kHz output). Evoked responses were recorded using the BioSemi ActiveTwo measurement system, with active electrodes at Cz, C7, and both mastoids. Common Mode Sense and Driven Right Leg electrodes were located on the low forehead and electrode offsets were maintained within ±40 mV throughout each recording. Bioelectric activity from each electrode was digitized at a sampling rate of 16384 Hz and processed off-line in MATLAB (The Mathworks Inc., 2013). EEG data files incorporated stimulus timing information by means of a custom trigger box connecting the external audio interface to the BioSemi USB interface.

#### 2.4.2 Auditory brainstem response

Digital stimuli were single-polarity high-pass filtered clicks (first-order butterworth, 2.4 kHz cutoff). Due to the low-pass response of the ER3A inserts, the stimuli in the ear canal had a 10 dB bandwidth extending from about 1.2 to 4.7 kHz (measured in a Gras IEC60711 occluded-ear simulator coupled to ER3A insert earphones). In order to minimize recording time, presentation alternated between ears, at a rate of 7.05 per second in each ear, so that a click in one ear was followed after approximately 71 ms by a click in the other ear. This gave an overall presentation rate of 14.1 per second and a total of 7040 presentations per ear. The inter-stimulus interval was jittered by a maximum of 10%, so as to prevent accumulation of stationary interference. In order to stimulate low-SR fibers, a presentation level of 102 dB peSPL (peak-to-peak) was selected, 2 dB higher than the maximum level used by Schaette and McAlpine (2011).

Activity between Cz and ipsilateral mastoid was filtered (30–1500 Hz; fourth-order butterworth) and divided into epochs extending from 10 ms pre-stimulus to 13 ms post-stimulus, after correcting for the 0.8 ms acoustic delay introduced by the sound tube. Post-hoc artifact rejection eliminated epochs whose RMS amplitude exceeded the mean by more than two standard deviations. The remaining epochs were averaged and corrected for any linear drift by subtracting a linear fit to the pre-stimulus baseline.

Waves I and V of the ABR were identified and quantified automatically in MATLAB (The Mathworks Inc., 2013), based on waveform characteristics within specified time windows. The window for wave I extended from 1.55 to 2.05 ms after stimulus peak and the window for wave V from 5.1 to 6.5 ms. The trough of wave I was required to occur 0.3 to 1.0 ms after its peak. The peak and trough of wave I were defined as local maxima and minima. Wave V required more subtle denotation, in order to appropriately interpret waveforms featuring a prominent wave IV or blended wave IV/wave V complex. Hence the peak of wave V was defined as either a local maximum or a downward inflection point on a falling portion of the waveform (a maximum in the first derivative where the first derivative < 0). Wave I amplitude was measured from peak to following trough. Wave V was measured from peak to baseline, in order to capture the gradual rise in amplitude from pre-stimulus baseline to wave V peak observed in all waveforms (presented in Supplementary Material). Post-hoc subjective review verified that all waveforms had been appropriately interpreted by the peak-picking algorithm. The resulting amplitudes and latencies were averaged across left and right ears for each participant.

#### 2.4.3 Envelope following response

**Fig. 2:**
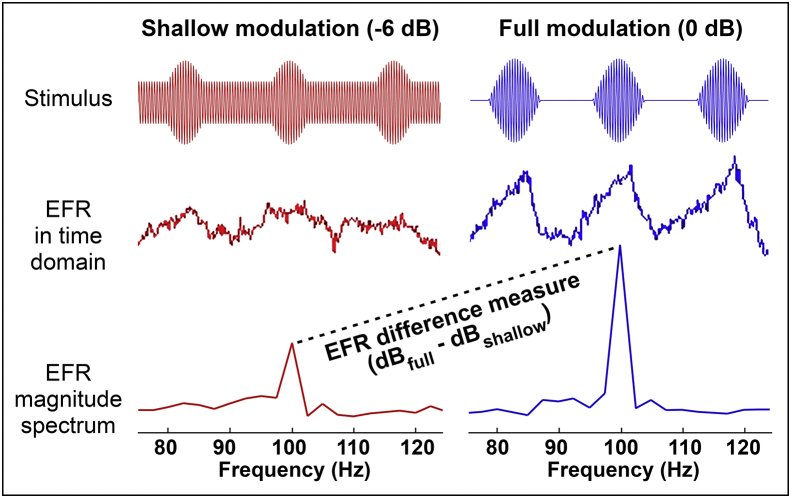
A schematic illustration of the EFR paradigm, including responses and response spectra from a single participant. Analyzed measures were the raw response amplitude at the frequency of interest, 100 Hz, and an EFR difference measure comparing response amplitudes at two stimulus modulation depths. It was predicted that loss of low-SR fibers should primarily impair responses at the shallow modulation depth, leading to higher values of the difference measure in synaptopathic ears.

Subcortical EFRs were recorded using the variable-modulation-depth paradigm described by Bharadwaj et al. (2015). Stimuli were 75 dB SPL transposed tones (Bernstein and Trahiotis, 2002) with a 4000 Hz carrier and 100 Hz modulator (Fig. 2). Stimulus duration was 400 ms with the addition of 15 ms onset and offset ramps. Off-frequency contributions were attenuated by notched-noise maskers (10–20000 Hz overall bandwidth, with a notch width of 800 Hz centered on 4000 Hz) applied at a signal-to-noise ratio (SNR) of 20 dB (broadband RMS). The noise was realized separately for each trial, rather than being frozen between trials. Stimuli were of two modulation depths (0 dB and −6 dB re: 100% modulation) and each was presented in two polarities. The resulting four stimuli were presented in the sequence: 0 dB; inverted 0 dB; −6 dB; and inverted −6 dB. The average inter-stimulus interval was 400 ms, jittered by up to 10%. This sequence was presented 630 times.

Activity in the vertical channel from Cz to C7 was divided into epochs extending from 4 to 404 ms after the end of the stimulus onset ramp. Post-hoc artifact rejection eliminated epochs whose RMS level exceeded the 99th percentile for the recording. The remaining epochs were averaged and the opposing-polarity averages added to give the response to the temporal envelope. Response spectra were analyzed to yield EFR amplitude at the 100 Hz modulation frequency, as well a measure equal to the difference in EFR amplitude (in dB) at the two stimulus modulation depths (Fig. 2). The EFR difference measure is closely related to that of Bharadwaj et al. (2015) - the slope of the function relating EFR amplitude to modulation depth - though slope was defined by a three-point function in the previous study. Unlike the other electrophysiological measures, the EFR difference measure was expected to *increase* due to synaptopathy, since ears with depleted low-SR fibers should exhibit particularly weak encoding of shallow modulations. In order to compute the difference measure for a given participant, significant 100 Hz EFR peaks were required in response to both modulation depths (defined as > 3 dB SNR, with noise being estimated from the mean amplitude in 10 adjacent frequency bins).

### 2.5 Statistical analysis

Statistical analysis was performed using R (R Core Team, 2015). All significance tests were conducted two-tailed. Data were checked for normality and homogeneity of variance prior to testing, and non-parametric tests applied where necessary. No data points were missing for any variable, therefore analyses were based on a total sample size N = 40, divided evenly between tinnitus and control groups. For supplemental sex-separated analyses, the four subgroups (tinnitus male, tinnitus female, control male, and control female) were each sized n = 10.

## 3 Results

### 3.1 Noise exposure history

**Fig. 3:**
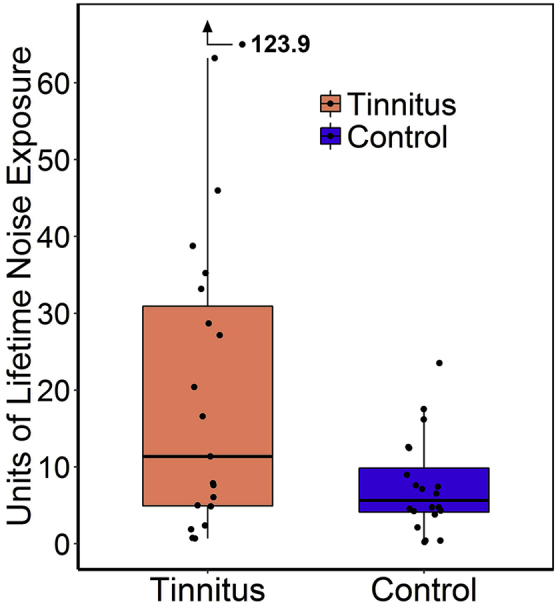
Units of lifetime noise exposure for participants in tinnitus and control groups. Points correspond to individual participants, upper and lower hinges to first and third quartiles, upper whiskers to the highest value within 1.5 * IQR of the upper hinge (where IQR is the interquartile range), and lower whiskers to the lowest value within 1.5 * IQR of the lower hinge.

Participants with TNA reported greater lifetime exposure than controls to sound levels over 80 dBA, Wilcoxon-Mann-Whitney U = 283, *p* = 0.02. However, as can be seen from Fig. 3, the spread of exposure values was greater for the TNA group, with some tinnitus participants presenting exposure scores in the same range as those of controls.

### 3.2 Auditory brainstem response

**Fig. 4:**
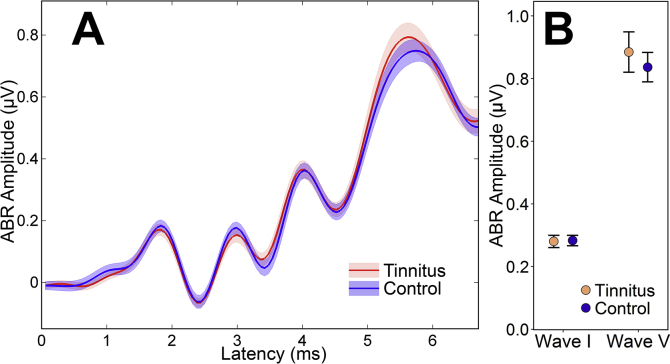
ABRs in response to 102 dB peSPL clicks for tinnitus and control groups. A: Grand average waveforms. Shaded areas correspond to the standard error of the mean. B: Wave I and wave V amplitudes, presented as mean ± standard error of the mean.

All participants produced unambiguous ABRs bilaterally, with waves I and V clearly evident at appropriate latencies. (Automatically interpreted waveforms are presented in Supplementary Material. Grand average waveforms are displayed in Fig. 4A.) Resulting amplitude and latency data are given in Table 4 (Supplementary Material).

As can be seen from Fig. 4B, the amplitude of ABR wave I was not significantly reduced in participants with tinnitus relative to controls, *t*(37.0) = −0.11, *p* = 0.91, Student's *t*-test. Note that had a one-tailed test been applied to these data, the result would have remained non-significant, *p* = 0.46. Measurement variability was low (coefficient of variation 0.26 in controls, 0.30 in tinnitus), giving statistical power of 90% (*α* = 0.05, one-tailed) to detect the 26% reduction in wave I amplitude for tinnitus versus controls reported by Schaette and McAlpine (2011) for a 100 dB peSPL click.

In an attempt to manage non-synaptopathic sources of variability in ABR amplitude, we computed the ratio of wave I to wave V amplitude, thought to provide a measure of central gain in the auditory brainstem (Schaette and McAlpine, 2011). This self-normalized difference measure did not differ significantly between groups, Wilcoxon-Mann-Whitney U = 192, *p* = 0.84. Nor did the amplitude of wave V, *t*(34.7) = 0.60, *p* = 0.55, Student's *t*-test. Supplemental sex-separated analyses revealed no significant effects of tinnitus on wave I amplitude (female *p* = 0.56, male *p* = 0.54, Student's *t*-tests) nor on wave I/V amplitude ratio (female *p* = 0.52, unequal variance *t*-test; male *p* = 0.44, Wilcoxon-Mann-Whitney test).

### 3.3 Envelope following response

**Fig. 5:**
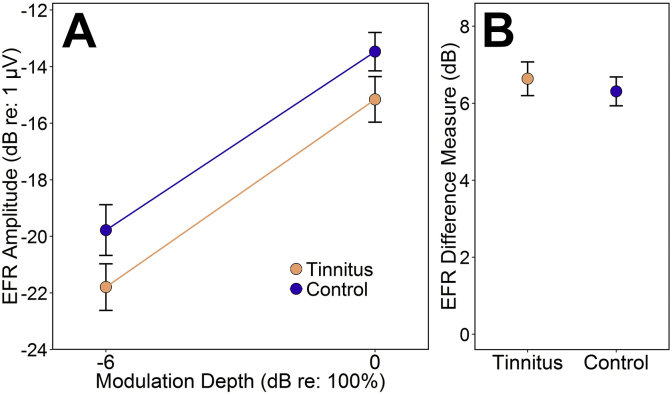
EFR measures for tinnitus and control groups, presented as group mean ± standard error of the mean. A: EFRs to transposed tones with a shallow (−6 dB) and full (0 dB) modulation depth. The tinnitus-related reduction in response amplitude is non-significant. The lines connecting the responses illustrate the “EFR slope” measure devised by Bharadwaj et al. (2015), though defined by a two-point function. B: The difference in EFR amplitude (in dB) at the two modulation depths. The hypothesized enhancement in the tinnitus group is not evident.

EFRs to stimuli of both modulation depths exceeded the noise floor for all participants, allowing analysis of both EFR amplitude (Fig. 5A) and the EFR difference measure (dB difference in response amplitude at the two modulation depths, Fig. 5B). The transposed tone with shallow modulations invariably elicited a lower EFR amplitude than the fully modulated stimulus, yielding consistently positive values of the EFR difference measure (see Table 5 of Supplementary Material). EFR amplitudes were entered into a two-way ANOVA, with tinnitus group as a between-subjects factor and stimulus modulation depth as a within-subject factor. There was a non-significant main effect of group, *F*(1,38) = 2.83, *p* = 0.10, with tinnitus subjects producing lower response amplitudes than controls. The absence of a significant interaction effect indicates that tinnitus is not significantly associated with differences in the EFR difference measure, *F*(1,38) = 0.324, *p* = 0.57. When the same analysis was performed on each sex separately, the results revealed no significant effects of tinnitus on EFR amplitude (male *p* = 0.29; female *p* = 0.23), nor significant interactions between group and depth (male *p* = 0.31; female *p* = 0.81).

### 3.4 Correlations between noise exposure and electrophysiological measures

**Fig. 6:**
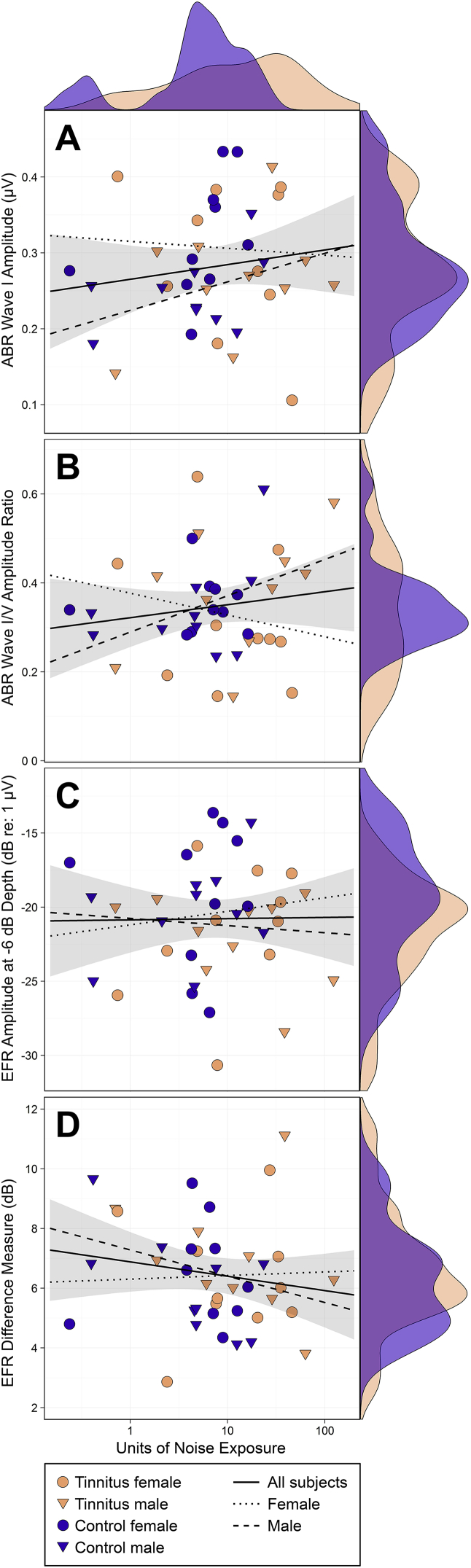
Relations between lifetime noise exposure and electrophysiological measures of cochlear synaptopathy, including both raw amplitude measures and self-normalized difference measures. Shaded areas represent 95% confidence limits of linear regression lines for all subjects. Marginal density plots represent tinnitus and control group distributions. No significant correlation is evident between noise exposure and any electrophysiological measure. A: ABR wave I amplitude. B: ABR wave I/V amplitude ratio. C: EFR amplitude at a shallow (−6 dB) modulation depth. D: Difference in EFR amplitude (in dB) at two stimulus modulation depths. Note that D was hypothesized to exhibit a positive relation, whereas negative relations were expected in A to C.

Pearson's product-moment correlation coefficients were computed to test the linear relations between log-transformed units of lifetime noise exposure and the various measures of neural function (Fig. 6). No association was evident between noise exposure and the amplitude of ABR wave I, *r* = 0.15, *p* = 0.36, nor between noise exposure and the ratio of wave I to wave V amplitude, *r* = 0.15, *p* = 0.35. Nor did noise exposure relate to EFR amplitude at a shallow modulation depth, *r* = 0.01, *p* = 0.94, or to the EFR difference measure, *r* = −0.16, *p* = 0.31. Note that in the latter case, it is predicted that the measure should increase with increasing noise exposure.

## 4 Discussion

### 4.1 A role for noise exposure in tinnitus with a normal audiogram

Reported lifetime noise exposure of tinnitus subjects exceeded that of controls, despite close matching on the basis of sex, age, and audiometric thresholds. To the authors’ knowledge, these data represent the first published evidence implicating noise exposure in tinnitus without threshold elevation. Previous research has associated excessive noise exposure and tinnitus in normally hearing young people (Davis et al., 1998, Meyer-Bisch, 1996) but not through comparison with audiometrically matched controls. Hence noise exposure in previous reports may have been related to tinnitus through sub-clinical threshold changes.

In contrast, our tinnitus group exhibited no significant reduction in hearing sensitivity at any of 10 measurement frequencies between 0.25 and 14 kHz. Though we cannot rule out the existence of narrow audiometric “notches” in our tinnitus subjects, undetected by standard audiometry (Zhao et al., 2014), these findings nonetheless cast new light on the hazards of noise to the auditory system. It seems that excessive noise exposure can induce changes in auditory function that spare the audiogram, even at high frequencies, and yet may lead to disturbing perceptual consequences.

### 4.2 No ABR evidence for tinnitus-related or noise-induced synaptopathy

**Fig. 7:**
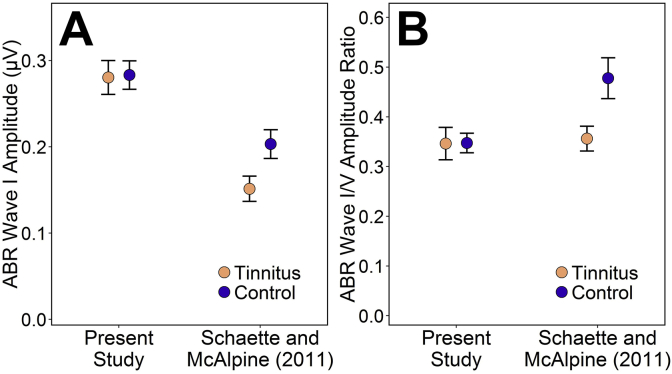
ABR data from the present study, elicited using 102 dB peSPL clicks, presented alongside those of Schaette and McAlpine (2011), elicited using 100 dB peSPL clicks. Points and error bars represent the mean ± standard error of the mean. A: The raw amplitude of ABR wave I. B: The ratio of wave I amplitude to wave V amplitude.

The nature of these noise-induced changes is very much less clear, since our measures revealed no evidence for cochlear synaptopathy in TNA. In particular, the expected reduction of ABR wave I amplitude was not observed. This finding stands in contrast with those of Schaette and McAlpine (2011), whose TNA subjects exhibited reduced wave I amplitudes relative to matched controls: reductions of 25% and 26% at 90 and 100 dB peSPL, respectively. Fig. 7 compares Schaette and McAlpine's 100 dB data with the data obtained in the present study.

Type II error is unlikely to account for these divergent findings, since post-hoc power analysis for the present study indicates 90% power to detect a 26% reduction in wave I amplitude (see Section 3.2). This is despite inclusion of participants of both sexes, which might reasonably be expected to increase ABR amplitude variability. The present study's wave I amplitude data are less variable than those of Schaette and McAlpine, perhaps due to the use of research-grade recording equipment. Therefore, other possible explanations for our null result must be considered.

It is plausible that differences in participant age between the two studies are responsible, an explanation which would have important implications for our understanding of both cochlear synaptopathy and tinnitus heterogeneity. Participants in the present study were considerably younger (mean tinnitus age 25.7 years, control 25.5 years) than those of Schaette and McAlpine (mean tinnitus age 36.3 years, control 33.2 years). It may be that cochlear synaptopathy is a significant etiology of TNA in older humans, but not among the very young, in whom other etiologies dominate.

It is therefore notable that evidence of human cochlear synaptopathy in relation to noise exposure is considerably less concrete than the evidence in relation to aging. Age-related loss of spiral ganglion cells was observed by Makary et al. (2011) in a large study of human temporal bones without significant hair cell loss. Parallel findings in mice (Sergeyenko et al., 2013) and preliminary synaptic counts in humans (Viana et al., 2015) strongly suggest that this decline is the delayed sequel to age-related cochlear synaptopathy progressing throughout the lifespan. In contrast, research relating human AN function to noise exposure has relied on electrophysiological measures, with mixed results. The results of the present study show no relation of lifetime noise exposure to ABR wave I amplitude, nor to ABR wave I/V amplitude ratio. Previously, Stamper and Johnson (2015a) reported a negative relation between noise exposure (estimated over the previous 12 months) and ABR wave I amplitude, but results were confounded by sex. Subsequent sex-separated analysis revealed that the correlation was present only in females in response to a 120 dB peSPL stimulus (Stamper and Johnson, 2015b). Using electrocochleography in college students, Liberman et al. (2016) found no significant association between reported noise exposure and the amplitude of the compound action potential (equivalent to ABR wave I), although a noise-related enhancement of the summating potential was observed. In a large study of 126 normally hearing young listeners, Prendergast et al. (2016) demonstrated no relation between lifetime noise exposure and wave I amplitude or EFR synchronization strength.

One explanation for this pattern of results is that audiometrically normal humans do not exhibit substantial synaptopathy solely as a result of noise exposure. Other possible explanations exist, such as insensitivity of electrophysiological measures (discussed later in Section 4.2) and diverse genetic susceptibility to synaptopathy in humans, who might have “tough” and “tender” ears (Henderson et al., 1993). However, it remains plausible that synaptopathy arises in humans due primarily to aging, or to an interaction between aging and noise exposure (as demonstrated in mice by Fernandez et al., 2015). This manifestation would represent a divergence from mouse models, but increasing evidence suggests that such inter-species differences are to be expected. Noise-induced synaptopathy in guinea pigs requires higher sound levels than in mice and long-term degeneration of spiral ganglion cells is less pronounced (Lin et al., 2011). In stark contrast with mouse data, guinea pig synapses damaged by noise appear largely repairable (Liu et al., 2012, Shi et al., 2013), leading to only transient changes in the distribution of spontaneous rates among AN fibers (Song et al., 2016). Early indications from a macaque model suggest that primates may exhibit even greater resistance to noise-induced synaptopathy (Burton et al., 2016).

Alternatively, it is conceivable that synaptopathy exists in audiometrically normal young humans, but is limited to extremely basal cochlear regions. This possibility is suggested by differences in ABR stimulus bandwidth between the present study and that of Schaette and McAlpine (2011). In order to limit the unwanted influence of very high frequency audiometric loss, we selected stimuli with a 10 dB bandwidth extending from 1.2 to 4.7 kHz. By comparison, our measurements indicate that the 10 dB bandwidth of Schaette and McAlpine's 100 dB clicks extends to 7.1 kHz (recorded in a Bruel and Kjaer 4153 artificial ear coupled to TDH-49 headphones). The high presentation level of our stimuli ought to elicit the “half-octave basalward shift” in the travelling wave, leading to strong excitation of characteristic frequencies up to approximately 7 kHz. With the addition of upward spread of excitation, the stimulated region should encompass the 3–6 kHz characteristic frequency region where early noise damage is usually manifest (Coles et al., 2000). Nevertheless, it remains possible that synaptopathy existed in our tinnitus cohort, but was restricted to even higher frequencies. Participants generally reported tinnitus with a high frequency percept and tinnitus pitch was not measured.

A crucial and related issue is that of high frequency audiometric loss and its influence on ABR wave I. It is possible that the ABR findings of Schaette and McAlpine (2011) and Gu et al. (2012) reflect basal loss of sensitivity in tinnitus participants, rather than an audiometrically “hidden” hearing loss. Failure to replicate these findings might indicate robustness of our methods against the unwanted influence of audiometric loss, given the audiometric and stimulus differences between the present study and the previous reports. Wave I of the ABR is dominated by contributions from high frequency portions of the cochlear partition, where reduced dispersion enhances the synchrony of neuronal firing (Don and Eggermont, 1978). At high stimulus levels, upward spread of excitation involves increasingly basal generators (Eggermont and Don, 1980). Hence the unambiguous interpretation of wave I amplitude may require careful control of audiometric thresholds at frequencies well beyond the bandwidth of the ABR stimuli. The present study used not only a narrower stimulus bandwidth than the previous studies, but closer audiometric matching (group means differed by < 1 dB at 10 and 14 kHz). Schaette and McAlpine's groups differed in audiometric sensitivity at 12 kHz, where mean threshold for the tinnitus group was 3.5 dB higher than for controls. Missing data (from five tinnitus subjects and three control subjects) prevented comparison at higher frequencies. Similarly, Gu et al. (2012) reported a significant reduction in wave I amplitude only for their 120 dB peSPL stimulus, for which missing ABR data led to systematic differences between groups in high frequency hearing sensitivity (tinnitus group had ∼ 10 dB higher thresholds at 14 kHz). The band-limited ABR stimuli used in these studies fall within the low-frequency tails of high-frequency AN fiber tuning curves, and hence the response of these fibers should be relatively unaffected by outer hair cell dysfunction at least (Liberman and Dodds, 1984). However, it remains possible that tinnitus-related ABR differences in previous reports were at least partially driven by basal loss of sensitivity.

Finally, it is worth considering that absence of ABR evidence for tinnitus-related synaptopathy might reflect insensitivity of the ABR rather than absence of synaptopathy. In addition to the variability of ABR amplitude, which has many sources and might obscure neuropathic effects, the findings of Bourien et al. (2014) cast doubt on the fundamental contribution of low-SR fibers to ABR wave I (see Section 1). Ongoing attempts to develop more sensitive electrophysiological measures of cochlear neuropathy are clearly warranted.

### 4.3 No EFR evidence for tinnitus-related or noise-induced synaptopathy

Several alternatives to the ABR have been proposed as viable measures of synaptopathy in humans, including the amplitude ratio of the compound action potential to the summating potential (Liberman et al., 2016) and round window neural noise (Batrel et al., 2016). Among them, the EFR has shown promise in both animals and humans and has the advantage of being recordable non-invasively, without the use of ear canal or transtympanic electrodes. However, the relation of the EFR to AN function is difficult to interpret, since contributions from different auditory centers are not separated in time as they are for the ABR, and the resulting response is dependent on neural function central to the AN. Additionally, and in common with the ABR, EFR amplitude reflects many non-synaptopathic sources of variability. Hence researchers have sought innovative EFR measures with enhanced sensitivity to synaptopathy. The difference measure devised by Bharadwaj et al. (2015) - the slope of the function relating EFR amplitude to stimulus modulation depth - was intended as a sensitive, self-normalized measure of low-SR fiber loss. EFR slope was shown to correlate with behavioral measures of temporal coding and auditory selective attention, with individual differences tentatively attributed to synaptopathy (Bharadwaj et al., 2015).

The present study utilized an EFR difference measure very closely related to that of Bharadwaj and colleagues: the difference in EFR amplitude (in dB) at two stimulus modulation depths. Many stimulus characteristics were also shared with the previous study: level, duration, carrier frequency, modulation frequency, and off-frequency masking characteristics. Yet this measure was not associated with tinnitus status, nor with lifetime noise exposure. These results might be taken to indicate lack of noise-induced or tinnitus-related cochlear synaptopathy in our cohort. However, it is also possible that this pathology is not, after all, a major source of individual differences in EFR slope. The hypothesized sensitivity of the measure to synaptopathy relies upon several assumptions, including preferential damage to low-SR fibers in humans and saturation of high-SR units by stimuli with shallow modulations. There is some evidence, for example, that the high-SR fiber dynamic range for modulated stimuli considerably exceeds that for steady-state stimuli (Smith and Brachman, 1980). Interpretation of the present results would be aided by validation of the EFR slope measure in an animal model of synaptopathy.

Methodological differences between the present study and that of Bharadwaj et al. (2015) are also to be considered, though they appear unlikely to compromise sensitivity. The earlier study computed slopes using a minimum modulation depth of −8 dB, employing multichannel recording and principal component analysis to enhance response SNR. The present study used a single channel and selected a −6 dB minimum modulation depth to ensure that all responses exceeded the noise floor. However, Bharadwaj and colleagues reported that temporal perceptual performance correlated not only with EFR slope but also with raw EFR amplitude for a −4 dB depth, implying that extremely shallow modulations were not an essential stimulus feature.

In addition to the EFR difference measure, the present study also analyzed straightforward EFR amplitude. EFR amplitude was not associated with lifetime noise exposure and did not differ significantly between tinnitus and control groups. Data from a mouse model indicate that EFR amplitude can be a robust measure of cochlear synaptopathy, but suggest that some features of our stimuli (and those of Bharadwaj et al., 2015) were suboptimal (Shaheen et al., 2015). The researchers used fully modulated EFR stimuli, optimized to enhance the contribution of the AN, and found that synaptopathy led to greater changes in EFR amplitude than in EFR phase locking value or ABR amplitude. Optimum sensitivity was achieved with high modulation frequencies (∼1 kHz), which limited the influence of more central nuclei. In contrast, the present study used a much lower modulation frequency and likely elicited the responses of higher centers, where the effects of deafferentation might be mitigated by enhanced central gain (Brotherton et al., 2015, Chambers et al., 2016). Hence the present EFR amplitude data must be interpreted with caution. The observed trend for lower amplitudes in TNA was not significant, but it is possible that stimuli with higher modulation rates might have been more effective in revealing AN temporal coding deficits. Future investigation of cochlear synaptopathy in humans might be well served by optimized EFR measures paralleling those applied successfully in rodent models.

### 4.4 Conclusions

The ABR and EFR results of the present study provide no evidence for cochlear synaptopathy in young humans with tinnitus and normal audiometric thresholds. Nor do these electrophysiological measures relate to lifetime noise exposure, providing no evidence for noise-induced synaptopathy in this cohort. It is importance to emphasize, however, that our results do not imply that synaptopathy is not prevalent in humans. It is possible, for example, that synaptopathy would have been measurable in an older population, through assessment of characteristic frequencies above 7 kHz, or through use of a more sensitive measure.

Tinnitus participants are, as a group, more noise exposed than controls, though also more heterogeneous in this regard. Uncertainty about mechanisms notwithstanding, the findings relating noise exposure and TNA have important implications. Even in tinnitus sufferers whose audiometric thresholds are indistinguishable from those of controls, symptoms may arise from sub-clinical damage due to excessive noise exposure.

## Figures and Tables

**Fig. s1:**
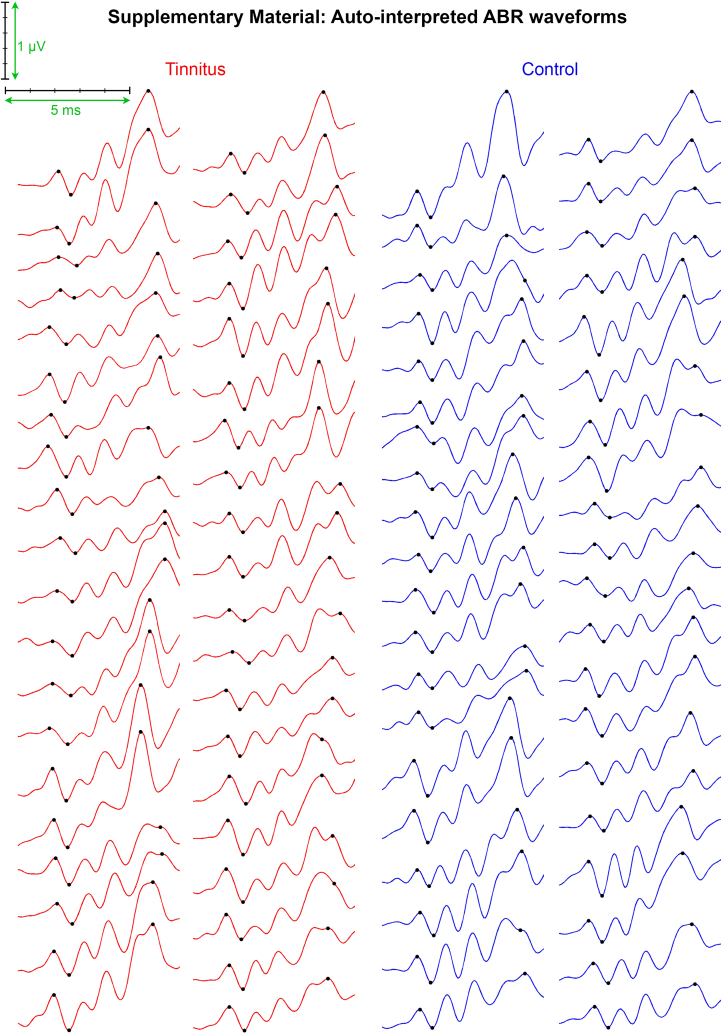


**Fig. s2:**
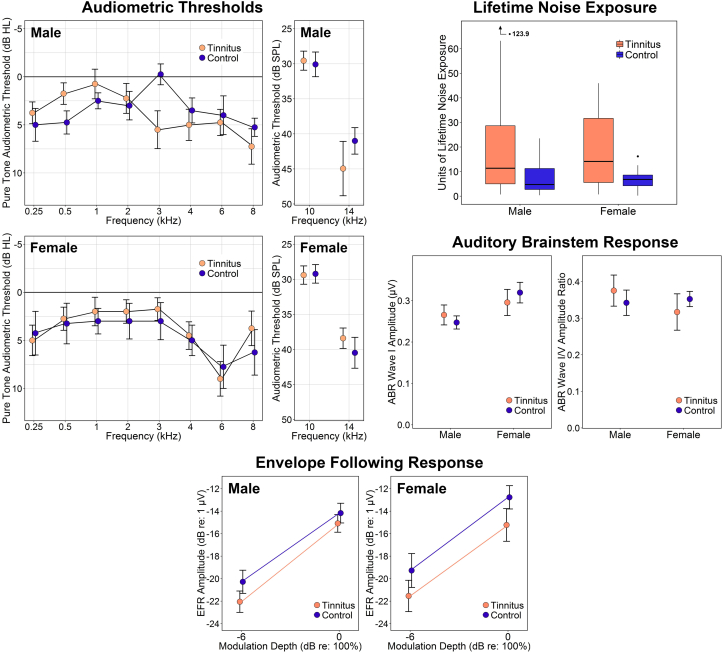


**Table 1:** Tinnitus characteristics.

ID	Tinnitus location	Sound quality	Time since onset	Constant in quiet?	TFI score	Conscious awareness of tinnitus (% of waking hours)
1	Both ears	Ringing	9 years	Yes	26.8	30
6	Right ear	Ringing	2 years	Yes	28	30
7	Both ears	High pitched whine	10 years	Yes	22.8	30
8	Both ears (right > left)	Between whining and ringing	> 6 years	Yes	8.4	50
9	Both ears (right > left)	Ringing	14 years	Yes	29.6	60
10	Both ears	Shooshing	> 12 years	Yes	6.4	40
12	Both ears	Ringing	14 years	Yes	20.8	30
19	Both ears (possibly right > left)	Buzzing	1 year	Yes	51.6	30
20	Both ears	High pitched tone	10 years	Yes	78	70
23	Both ears	Ringing	8 years	Yes	18	10
28	Both ears (left > right)	Ringing	2 years	Yes	32	20
29	Both ears (right > left)	Ringing	3 years	Yes	45.6	80
30	Central percept	Ringing or whining	1 year	Yes	48.4	50
32	Both ears	Ringing	8 years	Yes	23.6	40
34	Both ears	Ringing	Always	Yes	62	60
35	Both ears	Ringing	> 10 years	Yes	5.2	20
36	Both ears (left > right)	High frequency tone	7 years	Yes	24.4	30
37	Both ears	High pitched fridge noise	5 years	Yes	48	60
38	Can affect either ear	High pitched ringing	10 years	No (lasts minutes to hours)	71.6	60
57	Both ears (left > right)	Ringing	4 months	Probably, but uncertain	6.4	10
