# Supplementary material for: Tinnitus with a normal audiogram: Relation to noise exposure but no evidence for cochlear synaptopathy
Source: Hear Res. 2017 Feb;344:265–74. doi: 10.1016/j.heares.2016.12.002 (PMC5256478; doi:10.1016/j.heares.2016.12.002)
Supplement: Supplementary file 1 [file mmc1.docx]

**Supplementary Material: Tables 1, 2 and 3**

| **Table 1: Conversion of Noise Exposure Levels from Participant Estimate to dBA** | | | |
| --- | --- | --- | --- |
| ***Free-field exposures*** | | ***Personal music players*** | |
| ***Required vocal effort*** | ***Estimated level*** | ***Volume control setting*** | ***Estimated level*** |
| Normal voice at 1.2m | **< 80 dBA** | <70% of maximum | **< 80 dBA** |
| Raised voice at 1.2m | **87 dBA** | 70% of maximum | **82 dBA** |
| Loud voice at 1.2m | **90 dBA** | 80% of maximum | **88 dBA** |
| Very loud voice at 1.2m | **93 dBA** | 90% of maximum | **94 dBA** |
| Shouting at 1.2m | **99 dBA** | Maximum volume | **100 dBA** |
| Shouting at 0.6m | **105 dBA** |  | |
| Shouting at listener’s ear | **110 dBA** |  |  |

| **Table 2: ABR Amplitude and Latency** | | | | |
| --- | --- | --- | --- | --- |
|  | ***Amplitude (µV)*** | | ***Sex-separated Wave I Amplitude (µV)*** | |
|  | ***Wave I*** | ***Wave V*** | ***Male*** | ***Female*** |
| ***Tinnitus*** | **0.280**  ± 0.019 | **0.885**  ± 0.063 | **0.265**  ± 0.024 | **0.295**  ± 0.032 |
| ***Control*** | **0.283**  ± 0.016 | **0.836**  ± 0.046 | **0.247**  ± 0.016 | **0.319**  ± 0.025 |
|  | ***Amplitude Ratio*** | | ***Latency (ms)*** | |
|  | ***Wave I/Wave V*** | | ***Wave I*** | ***Wave V*** |
| ***Tinnitus*** | **0.346**  ± 0.032 | | **1.761**  ± 0.026 | **5.806**  ± 0.060 |
| ***Control*** | **0.347**  ± 0.019 | | **1.788**  ± 0.021 | **5.878**  ± 0.053 |

| **Table 3: EFR Amplitude and Slope** | | | |
| --- | --- | --- | --- |
|  | ***Amplitude (dB re: 1 µV)*** | | ***Slope***  ***(dB amplitude***  ***/ dB depth)*** |
|  | ***-6 dB depth*** | ***0 dB depth*** |  |
| ***Tinnitus*** | **-21.8** ± 0.8 | **-15.2** ± 0.8 | **1.11** ± 0.07 |
| ***Control*** | **-19.8** ± 0.9 | **-13.5** ± 0.7 | **1.05** ± 0.06 |
