# Supplementary material for: Tinnitus with a normal audiogram: Relation to noise exposure but no evidence for cochlear synaptopathy
Source: Hear Res. 2017 Feb;344:265–74. doi: 10.1016/j.heares.2016.12.002 (PMC5256478; doi:10.1016/j.heares.2016.12.002)
Supplement: Supplementary file 2 [file mmc2.pdf]

Supplementary Material: Tables 2, 3, 4 and 5

Table 2: Conversion of noise exposure levels from participant estimate to dBA			
Free-field exposures		***Personal music players***	
Required vocal effort	*Estimated level*	*Volume control setting*	*Estimated level*
Normal voice at 1.2m	**< 80 dBA**	<70% of maximum	**< 80 dBA**
Raised voice at 1.2m	**87 dBA**	70% of maximum	**82 dBA**
Loud voice at 1.2m	**90 dBA**	80% of maximum	**88 dBA**
Very loud voice at 1.2m	**93 dBA**	90% of maximum	**94 dBA**
Shouting at 1.2m	**99 dBA**	Maximum volume	**100 dBA**
Shouting at 0.6m	**105 dBA**		
Shouting at listener's ear	**110 dBA**		

Table 3: Noise exposure calculation for a single participant							
Activity	Bar work in nightclub	Festivals (acoustic)	Gigs (at 18 to 31)	Gigs (at 31 to 36)	Nightclubs (at 16 to 22)	Nightclubs (at 22 to 31)	Nightclubs (at 31 to 36)
Additional information	Music on throughout shift	15 one-day festivals (all in past 5 years)	~8 per year	~2 per year	~2 nights per week during term time	~4 nights per week, 40 weeks per year	Once every ~3 months
Noise level information	Shout at 0.6m	Talk very loudly at 1.2m	Shout at 0.6m	Shout at 0.6m	Shout at 1.2m	Shout at 1.2m	Shout at 1.2m
Estimated noise level (dBA)	**105**	**93**	**105**	**105**	**99**	**99**	**99**
Years	1	5	13	5	6	9	5
Weeks/year	52	3	8	2	40	40	4
Days/week	3	1	1	1	2	4	1
Hours/day	4	12	3	3	5	5	5
Total duration (hours)	**624**	**180**	**312**	**30**	**2400**	**7200**	**100**
Type of hearing protector	None	3M foam plugs	None	None	None	None	None
Protector attenuation (dB)		21					
Proportion of time worn		10%					
Units of noise exposure	**9.49**	**0.16**	**4.74**	**0.46**	**9.17**	**27.50**	**0.38**
TOTAL UNITS OF LIFETIME NOISE EXPOSURE = 51.89							

Table 4: ABR Amplitude and Latency

	Amplitude (µV)		Amplitude Ratio	Latency (ms)	
	Wave I	Wave V	Wave I/Wave V	Wave I	Wave V
Tinnitus	**0.280** ± 0.019	**0.885** ± 0.063	**0.346** ± 0.032	**1.761** ± 0.026	**5.806** ± 0.060
Control	**0.283** ± 0.016	**0.836** ± 0.046	**0.347** ± 0.019	**1.788** ± 0.021	**5.878** ± 0.053

	Sex-separated Wave I Amplitude (µV)		Sex-separated Wave I/V Amplitude Ratio	
	Male	Female	Male	Female
Tinnitus	**0.265** ± 0.024	**0.295** ± 0.032	**0.375** ± 0.042	**0.317** ± 0.050
Control	**0.247** ± 0.016	**0.319** ± 0.025	**0.342** ± 0.035	**0.352** ± 0.021

Table 5: EFR Amplitude and Difference Measure

	Amplitude (dB re: 1 µV)		EFR Difference Measure (dB)
	-6 dB depth	0 dB depth	
Tinnitus	**-21.8** ± 0.8	**-15.2** ± 0.8	**6.63** ± 0.44
Control	**-19.8** ± 0.9	**-13.5** ± 0.7	**6.31** ± 0.38

	Amplitude at -6 dB depth (dB re: 1 µV)		Amplitude at 0 dB depth (dB re: 1 µV)		EFR Difference Measure (dB)	
	Male	Female	Male	Female	Male	Female
Tinnitus	**-22.1** ± 0.9	**-21.5** ± 1.4	**-15.1** ± 0.8	**-15.2** ± 1.5	**6.96** ± 0.62	**6.31** ± 0.63
Control	**-20.3** ± 1.0	**-19.3** ± 1.5	**-14.2** ± 0.9	**-12.8** ± 1.0	**6.11** ± 0.54	**6.51** ± 0.54
